# Fine‐tuning the *P. pastoris* iMT1026 genome‐scale metabolic model for improved prediction of growth on methanol or glycerol as sole carbon sources

**DOI:** 10.1111/1751-7915.12871

**Published:** 2017-11-21

**Authors:** Màrius Tomàs‐Gamisans, Pau Ferrer, Joan Albiol

**Affiliations:** ^1^ Department of Chemical Biological and Environmental Engineering Universitat Autònoma de Barcelona 08193 Bellaterra (Cerdanyola del Vallès) Barcelona Spain

## Abstract

The methylotrophic yeast *Pichia pastoris* (*Komagataella* spp.) is widely used as cell factory for recombinant protein production. In the past recent years, important breakthroughs in the systems‐level quantitative analysis of its physiology have been achieved. This wealth of information has allowed the development of genome‐scale metabolic models, which make new approaches possible for host cell and bioprocess engineering. Nevertheless, the predictive accuracy of the previous consensus model required to be upgraded and validated with new experimental data sets for *P. pastoris* growing on glycerol or methanol as sole carbon sources, two of the most relevant substrates for this cell factory. In this study, we have characterized *P. pastoris* growing in chemostat cultures using glycerol or methanol as sole carbon sources over a wide range of growth rates, thereby providing physiological data on the effect of growth rate and culture conditions on biomass macromolecular and elemental composition. In addition, these data sets were used to improve the performance of the *P. pastoris* consensus genomic‐scale metabolic model iMT1026. Thereupon, new experimentally determined bounds, including the representation of biomass composition for these growth conditions, have been incorporated. As a result, here, we present version 3 (v3.0) of the consensus *P. pastoris* genome‐scale metabolic model as an update of the iMT1026 model. The v3.0 model was validated for growth on glycerol and methanol as sole carbon sources, demonstrating improved prediction capabilities over an extended substrate range including two biotechnologically relevant carbon sources.

## Introduction

*Pichia pastoris* (*Komagataella* spp.) has become one of the most commonly used hosts for recombinant protein production (Corchero *et al*., 2013; Gasser *et al*., 2013) including biopharmaceuticals (Martínez *et al*., 2012; Walsh, 2014). Since 1995, the number of genes heterologously expressed in this yeast has steadily increased (Bill, 2014). The establishment of *P. pastoris* as widely used cell factory has been supported by the development of improved high cell density operational strategies (Cos *et al*., 2006), synthetic biology tools, such as the availability of novel constitutive and inducible promoters (Prielhofer *et al*., 2013; Weinhandl *et al*., 2014), the application of novel genetic engineering techniques for its manipulation (Vogl *et al*., 2013; Weninger *et al*., 2015), as well as increased body of knowledge of *P. pastoris* at the genetic and physiological levels.

Moreover, progress in synthetic biology of this yeast has also opened the door towards utilizing this yeast as whole‐cell biocatalyst for non‐native value‐added metabolite production (Pscheidt and Glieder, 2008; Heyland *et al*., 2010; Cheng *et al*., 2014; Geier *et al*., 2015).

At an industrial scale, reduced cost of raw materials is as important as high production yields for cost‐effective processes (Kroll *et al*., 2010; Gustavsson and Lee, 2016). In addition, in order to optimize the metabolite biosynthesis process to obtain high yields, it is also important to select the most appropriate substrate (Goldman, 2010). In this context, glycerol is a by‐product in the conventional biodiesel production process and therefore represents an attractive opportunity for revalorization of an industrial waste stream, that is, for the development of a glycerol‐based integrated biorefinery concept (Kiss *et al*., 2015). Indeed, glycerol is becoming an attractive carbon source in fermentation processes to produce high added value compounds (Johnson and Taconi, 2007; Yang *et al*., 2012; Valerio *et al*., 2015). Furthermore, the reduction degree of glycerol (4.67) is different from that of glucose (4.0), and therefore, higher yields of certain secondary metabolites can be obtained from this compound (da Silva *et al*., 2009). Nonetheless, crude glycerol is far from being pure and contents several other compounds such as methanol (Posada *et al*., 2012). Methanol is usually toxic for microbes with the exception of methylotrophic microorganisms. Thus, subsequent purification and refinement steps should be applied to the raw glycerol if it has to be used by non‐methylotrophic organisms. On the other hand, methanol is also an increasingly interesting C1 compound as building block for value‐added compound biosynthesis (Schrader *et al*., 2009; Khosravi‐Darani *et al*., 2013; Nguyen *et al*., 2016). In this context, *P. pastoris* is able to efficiently use glycerol and/or methanol as energy and carbon sources (Solà *et al*., 2007; Çelik *et al*., 2008; Jordà *et al*., 2014). In addition, the most extensively used promoters for heterologous gene expression in *P. pastoris* (namely, P_*GAP*_, constitutive and P_*AOX*_, inducible) are directly associated with glycerol and methanol metabolism (Cos *et al*., 2006; Gasser *et al*., 2013). Therefore, *P. pastoris* appears as an organism of high potential for the development of the glycerol biorefinery concept.

Genome‐scale metabolic models (GSMM) allow to predict the phenotype of a microorganism in a range of conditions, including those derived from genetic modification (Oberhardt *et al*., 2009; Kim *et al*., 2012). This capability makes GSMM a powerful tool for the design of metabolic engineering strategies to enhance productivities or implementing new pathways (Cvijovic *et al*., 2011; Gustavsson and Lee, 2016). Nevertheless, validation of GSMM for different conditions requires the availability of extensive cultivation data information describing its physiology. In addition, a wide range of information on biomass composition enables building specific biomass equations to accurately describe cell growth in each case (Dikicioglu *et al*., 2015).

Three independent GSMM for *P. pastoris* were initially published, namely *iPP*668 (Chung *et al*., 2010), PpaMBEL1254 (Sohn *et al*., 2010) and iLC915 (Caspeta *et al*., 2012). More recently, the consensus model iMT1026 has been published (Tomàs‐Gamisans *et al*., 2016), integrating and upgrading the previous models. The consensus iMT1026 model showed a significant improvement in prediction accuracy and was validated for two sets of conditions: growth on glucose as a sole carbon source under different oxygen availability conditions and growth on different glycerol and methanol mixtures as carbon sources at different growth rates. However, given the impact of biomass composition on the model predictive accuracy in a context‐dependent manner (Dikicioglu *et al*., 2015), this model was still not suitable for describing growth on glycerol or methanol as single carbon sources. This is because biomass composition equations take a major role on prediction reliability, and small changes in that composition, or using an inadequate biomass equation, may rend model calculations inaccurate (Dikicioglu *et al*., 2015). Hence, GSMMs are in continuous evolution (e.g. for *Saccharomyces cerevisiae* (Aung *et al*., 2013)) usually involving error‐fixing steps related to poor or wrong gene annotation (Dikicioglu *et al*., 2014), or extending GSMM capabilities for a broader range of cultivation conditions.

In this work, we expand the iMT1026 model capabilities by implementing the capacity of accurately describing *P. pastoris* growth phenotype when using glycerol or methanol as sole carbon sources.

A series of chemostat cultures were performed at a wide range of growth rates using glycerol or methanol as sole carbon sources in order to provide comprehensive physiological data sets needed to upgrade the model. This included quantitative analyses of the elemental and macromolecular biomass composition for each tested growth condition. This allowed to introduce new biomass reaction equations to the metabolic model specific for growth on glycerol or methanol. Furthermore, the new version of the model (v3.0) was validated for growth on these two substrates within the tested growth rate range.

## Results and discussion

### Physiological macroscopic parameters

*Pichia pastoris* X‐33 strain was cultivated in carbon‐limited chemostat cultures at different dilution rates to characterize its physiology using different carbon sources. This information was used to estimate the energetic parameters and to calibrate the model for such carbon sources. Glycerol cultivations were carried out at different dilution rates (D): 0.035, 0.050, 0.065, 0.100, 0.130 and 0.160 h^−1^. At 0.160 h^−1^, the inflowing gas was supplied with an air:O_2_ mixture (92.5:7.5) due to the higher O_2_ demand and cell concentration. Due to this operational limitation, no higher dilution rates were tested, despite *P. pastoris* has been reported to grow at higher growth rates (Cos *et al*., 2006). Methanol limiting chemostats were run at 0.035, 0.050, 0.065, 0.080, 0.100 and 0.130 h^−1^. At 0.130 h^−1^, bioreactor washed out. Biomass concentration, CO_2_ production and O_2_ consumption continuously decreased, and methanol accumulated. According to a chemostat washout kinetics (Doran, 1995), maximum growth rate on methanol was estimated to be between 0.11 and 0.12 h^−1^, which is in agreement with previously reported values (Barrigon *et al*., 2015).

Based on the chemostat data, specific productivities and yields were calculated for each condition (Table 1). In both glycerol and methanol cultivation series, main growth parameters show a linear correlation with growth rate (μ).

**Table 1:** Macroscopic growth parameters after the reconciliation procedure for glycerol and methanol cultivations at different dilution rates

Carbon source	μ_SP_ (h^−1^)	μ_exp_ (h^−1^)	q_S_ (mmol · gDCW^−1^ · h^−1^)	q_O2_ (mmol · gDCW^−1^ · h^−1^)	q_CO2_ (mmol · gDCW^−1^ · h^−1^)	q_X_ (Cmmol · gDCW^−1^ · h^−1^)	Y_XS_ (g_X_ · g_S_^−1^)	RQ
Glycerol	0.035	0.035 ± 0.001	−0.58 ± 0.05	−0.82 ± 0.13	0.53 ± 0.11	1.22 ± 0.04	0.65 ± 0.03	0.64 ± 0.03
	0.050	0.049 ± 0.002	−0.85 ± 0.06	−1.26 ± 0.10	0.84 ± 0.08	1.70 ± 0.14	0.62 ± 0.05	0.67 ± 0.07
	0.065	0.064 ± 5e‐4	−1.07 ± 0.01	−1.52 ± 0.02	1.00 ± 0.02	2.22 ± 0.02	0.64 ± 5e‐4	0.65 ± 5e‐4
	0.100	0.094 ± 0.004	−1.52 ± 0.08	−2.04 ± 0.11	1.28 ± 0.08	3.27 ± 0.15	0.71 ± 0.04	0.63 ± 0.01
	0.130	0.124 ± 0.001	−1.92 ± 0.08	−2.36 ± 0.15	1.41 ± 0.13	4.36 ± 0.24	0.71 ± 0.04	0.60 ± 0.07
	0.160	0.154 ± 0.002	−2.41 ± 0.03	−2.92 ± 0.01	1.74 ± 1e‐3	5.47 ± 0.08	0.70 ± 2e‐3	0.60 ± 3e‐3
	Average						0.67 ± 0.04	0.63 ± 0.03
Methanol	0.035	0.035 ± 0.001	−2.81 ± 0.16	−2.98 ± 0.22	1.59 ± 0.14	1.22 ± 0.02	0.38 ± 0.01	0.53 ± 0.01
	0.050	0.049 ± 2e‐4	−3.88 ± 0.10	−4.07 ± 0.15	2.15 ± 0.10	1.73 ± 0.01	0.39 ± 0.01	0.53 ± 0.01
	0.065	0.065 ± 0.001	−4.87 ± 0.22	−4.97 ± 0.29	2.55 ± 0.18	2.33 ± 0.04	0.41 ± 0.01	0.51 ± 0.01
	0.080	0.084 ± 0.001	−6.23 ± 0.16	−6.36 ± 0.18	3.27 ± 0.12	2.96 ± 0.13	0.42 ± 0.02	0.51 ± 0.02
	0.100	0.099 ± 0.001	−7.82 ± 0.28	−8.22 ± 0.37	4.34 ± 0.24	3.47 ± 0.04	0.40 ± 0.01	0.53 ± 0.01
	Average						0.40 ± 0.01	0.52 ± 0.01

μ_SP_ corresponds to the set point growth rate and μ_exp_, the measured experimental μ.

Regarding biomass yields (Y_XS_), there is a slight decrease at lower growth rates on both carbon sources, similarly as reported by Van Dijken *et al*. (1976) and Rebnegger *et al*. (2014). Despite this apparent correlation, there are no statistically significant differences within the tested range, and average Y_XS_ and RQ can be calculated for the abovementioned range of growth rates. Average Y_XS_ in methanol is 0.40 g_X_ · g_S_^−1^ and is in accordance with yields previously reported for *P. pastoris* and other yeast (Hazeu and Donker, 1983). This value is considerably lower than 0.67 g_X_ · g_S_^−1^, the average Y_xs_ for glycerol. The Y_XS_ for glycerol ranged between 0.62 and 0.71 g_X_ · g_S_^−1^, similar to yields on this substrate reported for different *Pichia* species and other yeasts (Taccari *et al*., 2012).

### Macromolecular and elemental biomass composition

#### Growth rate‐dependent stoichiometry

**Figure 1:**
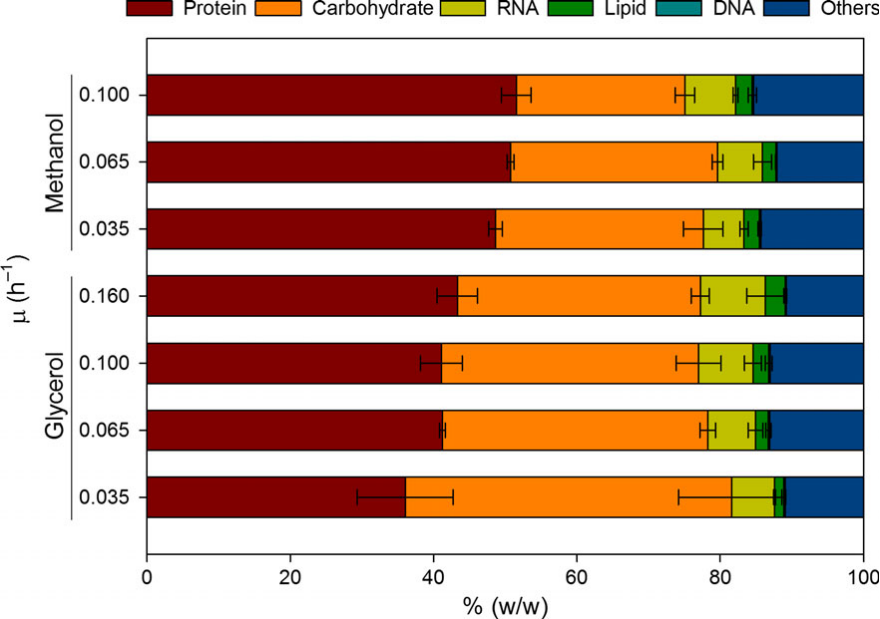
Comparison of the reconciled macromolecular composition of glycerol and methanol cultures at different growth rates.

To investigate the potential impact of growth rate on biomass composition, samples of the cultures were taken for analysis of the biomass elemental and macromolecular composition at different dilution rates. In particular, we analysed the biomass composition at four different growth rates for glycerol (μ = 0.035, 0.065, 0.100 and 0.160 h^−1^) and three for methanol (μ = 0.035, 0.065 and 0.100 h^−1^). The experimental data sets and the calculated (reconciled) biomass composition are summarized in Fig. 1 and Table 2.

**Table 2:** Detailed reconciled elemental and macromolecular composition of cells grown on glycerol and methanol at different growth rates, and the averaged biomass composition used for defining the stoichiometric coefficients in iMT1026 v3.0. Values represent weight/weight % ± SD

	Glycerol				Methanol			Average glycerol^a^	Average methanol^a^	Glucose^b^
D (h^−1^)	0.035	0.065	0.100	0.160	0.035	0.065	0.100			
Protein	36.0 ± 6.7	41.2 ± 0.4	41.1 ± 2.9	43.3 ± 2.8	48.6 ± 1.0	50.7 ± 0.5	51.5 ± 2.1	41.0 ± 1.5	50.1 ± 0.8	37.0 ± 2.4
Carbohydrate	45.6 ± 7.5	37.0 ± 1.1	35.9 ± 3.1	33.9 ± 1.3	29.0 ± 2.8	28.9 ± 0.8	23.5 ± 1.3	35.9 ± 2.0	27.3 ± 1.5	36.9 ± 3.5
Lipid	1.3 ± 0.3	1.8 ± 0.4	2.2 ± 0.5	2.8 ± 3e‐2	2.2 ± 0.2	1.9 ± 0.1	2.4 ± 0.6	2.5 ± 0.4	2.0 ± 0.2	6.2 ± 3.3
RNA	6.0 ± 0.1	6.7 ± 1.1	7.6 ± 1.2	9.1 ± 2.6	5.7 ± 0.6	6.3 ± 1.3	7.1 ± 0.4	7.8 ± 0.6	7.0 ± 0.6	6.6 ± 0.7
DNA	0.19 ± 1e‐3	0.19 ± 0.01	0.18 ± 0.01	0.18 ± 0.01	0.19 ± 4e‐3	0.18 ± 4e‐3	0.18 ± 0.02	0.19 ± 0.01	0.18 ± 0.01	0.13 ± 0.01
SO_4_	0.28 ± 0.06	0.40 ± 1e‐3	0.45 ± 0.10	0.46 ± 0.11	0.63 ± 4e‐3	0.69 ± 0.05	0.66 ± 0.06	0.46 ± 0.08	0.63 ± 0.04	0.3 ± 0.3
H_2_O	5.8 ± 0.6	5.7 ± 0.2	6.6 ± 2.5	6.6 ± 0.2	8.4 ± 0.1	6.2 ± 1.8	8.1 ± 0.7	5.6 ± 0.7	7.2 ± 0.9	6.3 ± 2.4
Metals	5.3 ± 1.0	7.0 ± 0.1	6.0 ± 0.9	5.8 ± 1.1	5.3 ± 2.1	5.2 ± 2.0	6.6 ± 0.1	7.0 ± 0.6	6.3 ± 0.6	6.4 ± 0.4
C	42.3 ± 0.1	41.9 ± 0.1	41.9 ± 0.9	42.44 ± 1.00	42.2 ± 0.8	43.2 ± 0.1	41.8 ± 0.2	41.98 ± 0.27	42.28 ± 0.53	43.0 ± 1.4
H	6.3 ± 3e‐2	6.24 ± 2e‐3	6.3 ± 0.2	6.44 ± 0.08	6.6 ± 0.1	6.5 ± 0.2	6.5 ± 0.1	6.24 ± 0.06	6.43 ± 0.05	6.3 ± 0.2
N	7.4 ± 1.2	8.4 ± 0.1	8.7 ± 0.7	9.22 ± 0.24	9.7 ± 0.1	10.2 ± 0.1	10.4 ± 0.4	8.58 ± 0.31	10.06 ± 0.16	6.9 ± 0.4
O	37.8 ± 2.2	35.4 ± 4e‐2	36.0 ± 2.6	34.98 ± 0.40	35.2 ± 1.3	33.9 ± 1.6	33.4 ± 0.7	35.11 ± 0.58	33.83 ± 0.46	36.4 ± 1.4
S	0.25 ± 0.06	0.30 ± 1e‐3	0.25 ± 0.04	0.30 ± 0.04	0.41 ± 0.01	0.4 ± 0.02	0.43 ± 0.03	0.30 ± 0.03	0.41 ± 0.01	0.2 ± 0.1
Ashes	5.9 ± 1.0	7.7 ± 4e‐3	6.8 ± 1.1	6.6 ± 1.3	5.9 ± 2.0	5.9 ± 1.8	7.4 ± 0.1	7.8 ± 0.6	7.0 ± 0.6	7.1 ± 0.4

a Average compositions are weighted averages using 1/SD.

b Corresponding to *P. pastoris* growing at *D *=* *0.1 h^−1^. Data taken from Carnicer *et al*. (2009)**.**

Notably, the protein and RNA fractions positively correlate with growth rate in both glycerol‐ and methanol‐fed cultivation series. This increment on protein and RNA with increasing growth rates is at expenses of carbohydrate content. This trade‐off between RNA–protein and carbohydrate content has been widely reported in yeast species (Verduyn *et al*., 1990; Verduyn, 1991), including in *P. pastoris* (Jordà *et al*., 2014). The increase in protein fraction is consistent with the measured changes in the elemental composition: the nitrogen content is also higher at high growth rates (Table 2). Nonetheless, only the correlation of RNA and growth rate is statistically significant. This stoichiometric dependence of biomass components on growth rate can be described on the basis of the growth rate hypothesis (GRH). Essentially, GRH attributes this shift to the tight control of the expensive protein synthesis system (Henriksen *et al*., 1996). At higher growth rates, cells need a higher ribosomal content to maintain the enzymatic machinery. The ribosomes are reported to consist of 53% RNA and 47% protein in *Aspergillus niger* (Hangeraaf and Muller, 2001). Thus, the increase in ribosome levels has a deep impact in overall cell protein and RNA content. As mentioned above, the increase in protein and RNA would be at expenses of the carbohydrate content. Biomass characterization in *S. cerevisiae* showed similar results, with a decrease in carbohydrate content at higher growth rates (Küenzi and Fiechter, 1972; Lange and Heijnen, 2001). At low growth rates, there is a larger fraction of carbon source not used for energy or cell machinery (protein/RNA) generation which is stored in the form of carbohydrates. As growth rate increases more and more, carbon source is derived towards energy and biosynthetic machinery generation at the expense of stored carbohydrates (Pejin and Razmovski, 1993).

Regarding the lipid fraction, no statistically significant differences were found across the series of methanol biomass samples collected at different growth rates. Conversely, the cell lipid fraction shows a positive correlation with the growth rate in glycerol‐grown cells. Nevertheless, a negative correlation of lipid content with growth rates has been commonly reported (Meeuwse *et al*., 2011; Rakicka *et al*., 2015). Therefore, the positive correlation observed in our case may be attributable to the strong reduction in relative carbohydrate content, which seems to be not entirely compensated by the increase in RNA and protein content.

#### Carbon source effects on biomass composition

Besides the impact of the specific growth rate on biomass composition described above, other factors such as the carbon source are also known to have a significant influence (Jordà *et al*., 2014). In our case, the effect of the carbon source can be appreciated in Fig. 1: cells grown on methanol show a significantly higher protein fraction than those grown on glycerol. This protein fraction is also higher than the one described for glucose‐grown cells (Table 2). However, similar profiles are observed when comparing glycerol‐specific biomass composition to the original biomass composition for glucose‐grown cells (Table 2) previously reported by Carnicer *et al*. (2009). Indeed, none of the macromolecular components of the glucose‐grown biomass showed any significant difference with the glycerol‐grown biomass in terms of relative abundances. In contrast, growth on methanol has a higher impact on the relative abundance of macromolecules, mainly increasing the protein fraction. This effect was also observed by Jordà *et al*. (2014) in a study where *P. pastoris* was grown in chemostats using different glycerol:methanol mixtures as carbon source. The corresponding biomass composition analyses showed that protein content increased when the methanol/glycerol ratio was higher. Similarly, *P. pastoris* cells growing on a glucose:methanol mix in chemostat cultivations showed higher protein content than when growing on glucose as a sole carbon source under analogous conditions (Jordà *et al*., 2012). Consequently, the increase in cell protein content seems to be directly related to methanol utilization and, more specifically, to the amount of enzymes needed for methanol assimilation (Rußmayer *et al*., 2015). In fact, it is known that genes encoding for the methanol utilization pathway such as the alcohol oxidase (*AOX*) and dihydroxyacetone synthase (*DAS*), two major enzymes involved in the initial steps of methanol metabolism, are highly induced in the presence of methanol (Rußmayer *et al*., 2015). They are reported to account for up to 10–20% of total protein in methylotrophic yeasts (Van Dijken *et al*., 1976; Stewart *et al*., 2001). This fact, together with the significant increase in the cell volume occupied by peroxisomes in methanol‐grown cells, may be a plausible explanation of the increase in cell protein content in these conditions (van der Klei *et al*., 2006; Veenhuis and van der Klei, 2014).

**Figure 2:**
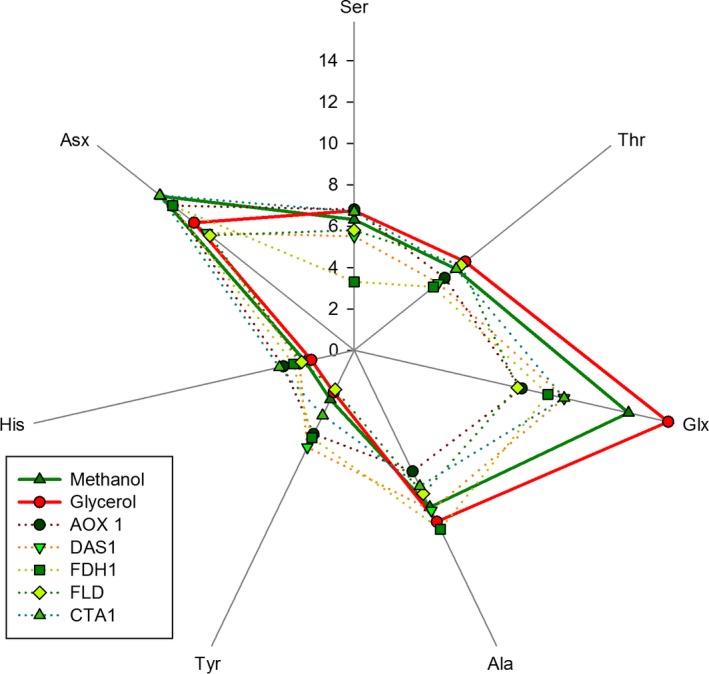
Comparison of average amino acid profiles from glycerol and methanol cultures in relation to amino acid abundance in the most abundant proteins in methanol metabolization. Amino acid abundance is presented as mol/mol %. Glycerol and methanol represent the average amino acid composition of glycerol and methanol cultivations respectively. Other variables correspond to the most abundant proteins in the presence of methanol: alcohol oxidase (AOX1), dihydroxyacetone synthase (DAS1), formate dehydrogenase (FDH1), formaldehyde dehydrogenase (FLD), catalase (CTA1). Glx and Asx represent the pair of Asp/Asn and Glu/Gln respectively.

On the other hand, amino acid composition analysis of the cell proteome showed no significant differences when comparing cells grown at different growth rates for each substrate Table S1 (Appendix S1). However, the amino acid composition of biomass differed significantly for some amino acids when comparing glycerol‐ versus methanol‐grown cells (Table S1). In addition, the subset of amino acids showing significant differences of relative abundances in methanol‐grown cells (compared to the glycerol condition) was compared with the amino acid composition of enzymes related to the methanol metabolization pathway (Fig. 2). This analysis clearly reveals how the amino acid composition of the methanol metabolization enzymes affects the overall cell amino acid composition with respect to glycerol. Therefore, the higher protein fraction in biomass composition in methanol appears to be related to the increased content of methanol‐assimilating pathway enzymes.

**Figure 3:**
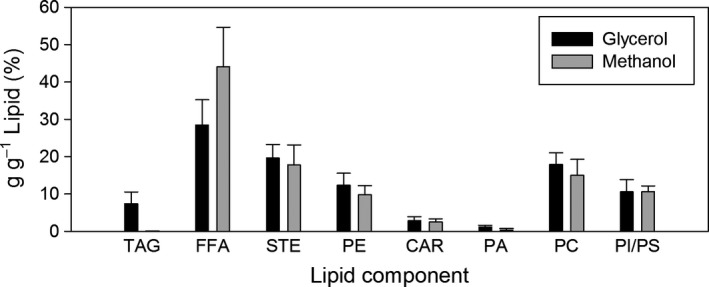
Average lipid profile for biomass grown on glycerol (black) and methanol (grey). Triacylglycerols (TAG), free fatty acids (FFA), sterols (STE), cardiolipin (CAR), phosphatidic acid (PA), phosphatidylcholine (PC) and phosphatidylinositol/phosphatidylserine (PI/PS).

In terms of cell total lipid content, no statistically significant differences were found when comparing the average carbon source‐specific biomass compositions. In addition, there are neither differences with previously described lipid fractions for cells grown on glucose nor with those grown in glucose–methanol mixtures (Carnicer *et al*., 2009; Jordà *et al*., 2014). Nevertheless, there are significant differences in the lipid composition profile of cells depending on the carbon source (Fig. 3, Table S2 in Appendix S1). Specifically, these differences are found in triacylglycerols (TAG), free fatty acids (FFA) and phosphatidic acid (PA). There is a higher content of TAG and PA at expenses of FFA in glycerol‐grown cells, whereas in methanol‐grown cells, FFA is the major lipid fraction, and TAG and PA are present only in trace amounts. Glycerol is a direct precursor for many lipids. In addition, the relative content of both TAG and PA, which are lipid molecules with a glycerol backbone, is increased in glycerol‐grown cells. Therefore, these differences seem to reflect the lower synthesis cost of TAG and PA from its direct precursor glycerol.

When formulating a biomass equation for glycerol and methanol growth conditions, despite that certain biomass components appear to be correlated with biomass‐specific growth rate, statistical analyses do not show significant differences associated with growth rate. In contrast, statistically significant differences are found when comparing average glycerol and methanol biomass compositions. Consequently, new biomass equations have been formulated for growth on glycerol and methanol incorporating specific equations for each relevant macromolecule (proteins, lipids) as well as for the fractional contribution of each macromolecule to biomass. The coefficients for the biomass equations were directly extracted from the average carbon source‐specific compositions reported in Table 2.

#### Energetic parameters estimation

Prior to model validation, energetic parameters have to be estimated in order to assure accurate predictions of cell performance. These parameters are the growth associated and the non‐growth associated maintenance energy (GAME and NGAME respectively). NGAME values differed significantly for glycerol and methanol growth conditions. On the one hand, growth on glycerol showed a NGAME of 2.51 mmol ATP · g_DCW_^−1^ · h^−1^, which is similar to the corresponding value previously calculated for glucose growth conditions, 2.81 mmol ATP · g_DCW_^−1^ · h^−1^ (Rebnegger *et al*., 2016; Tomàs‐Gamisans *et al*., 2016). In contrast, the NGAME calculated for methanol growth is 0.44 mmol ATP · g_DCW_^−1^ · h^−1^, i.e. much lower compared with the corresponding values calculated for the other carbon sources.

**Figure 4:**
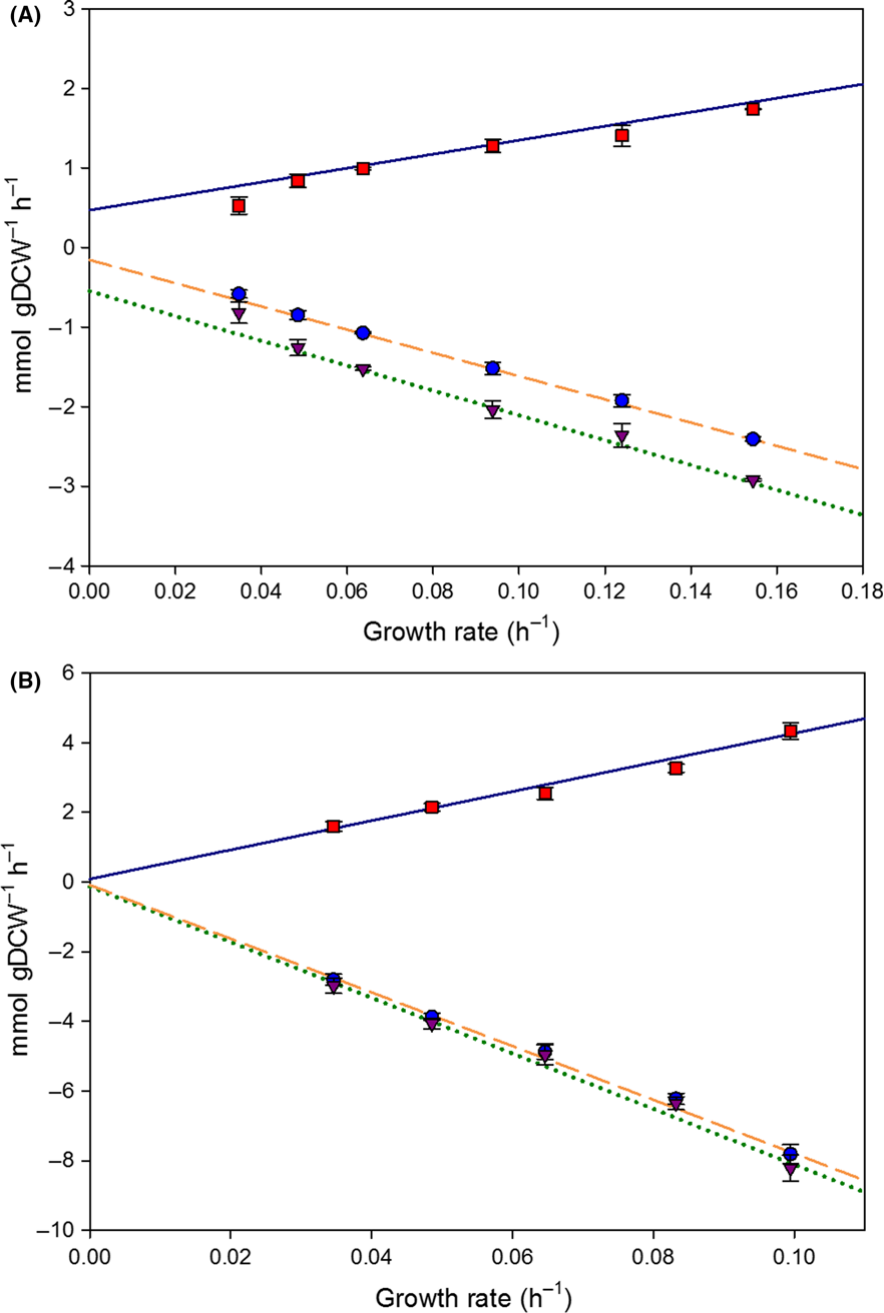
Evaluation of simulated and experimental macroscopic variables for the growth in glycerol and methanol. For each carbon source, the growth rate was constrained, and the absolute value of substrate uptake rate was minimized. A. Chemostats on glycerol. B. Chemostats on methanol. q_S__,__glycerol/methanol_ (●), q_CO__2_ (■), q_O__2_ (▼); predicted q_S__,__glycerol/methanol_ (dashed line), predicted q_CO__2_ (continuous line), predicted q_O__2_ (dotted line).

For GAME estimation for growth on glycerol, physiological parameters corresponding to the μ = 0.035 h^−1^ condition were not considered, as a metabolic shift seems to change the phenotypic profile at this (and lower) growth rates (Rebnegger *et al*., 2014). This can be directly inferred from the specific CO_2_ production rate (q_CO2_) and specific O_2_ consumption rate (q_O2_) observed at this growth rate, which do not follow the same linear trend as in the rest of measured range (Fig. 4). Hence, taking into account this consideration, GAME for glycerol was estimated to be 70.66 mmol ATP · g_DCW_^−1^, that is, 2.4‐fold lower than for methanol (166.77 mmol ATP · g_DCW_^−1^). As mentioned above, there is an important change in protein composition in methanol‐grown cells compared to glycerol growth due to the high levels of enzymes associated with methanol metabolization. The metabolic overload resulting from the maintenance of this cell machinery could be one of the reasons for the higher GAME besides the fact that growth on highly reduced substrates such as methanol (reduction degree (RD) of 6) is known to be usually less efficient (higher energy dissipation and lower biomass yields) compared to glycerol (RD 4.67) or glucose (RD 4; Heijnen and Van Dijken, 1992). When compared to glucose culture conditions, GAME for glycerol growth is very similar to the 72 mmol ATP · g_DCW_^−1^ calculated for glucose growth in our previous study (Tomàs‐Gamisans *et al*. (2016)).

#### Model validation

The updated model, iMT1026 v3.0 (Appendix S3 and available at BioModels Database with model ID MODEL1612130000), integrating the new specific biomass equations for growth on glycerol and methanol as sole carbon sources, was used to estimate the main macroscopic growth parameters as described in *Experimental procedures* section. The version 3.0 of iMT1026 accurately predicts macroscopic growth parameters within the range of tested growth rates for both carbon sources (Fig. 4).

Despite the great overall performance, model deviates from the experimental data by overestimating q_O2_ and q_CO2_ in the case of glycerol growth at 0.035 h^−1^ (Fig. 4). *P. pastoris* has been reported to reduce maintenance energy requirements at very low growth rates associated with metabolic adaptations and changes in gene expression (Rebnegger *et al*., 2014, 2016). To take into account this lower maintenance energy requirement, a series of additional simulations were carried out by constraining the NGAME at values lower than 2.51 mmol ATP g_DCW_^−1^ · h^−1^ (i.e. the default value set for glycerol‐grown cells) and maximizing growth at a given substrate uptake rate. In this way, iMT1026 v3.0 can be used to accurately predict the main macroscopic growth parameters for glycerol growth at 0.035 h^−1^ when NGAME is lowered (Fig. S1 in Appendix S1). In particular, values between 1 and 1.5 mmol ATP g_DCW_^−1^ · h^−1^ allow the best accuracy in predicting the experimental data at 0.035 h^−1^, as shown in Fig. S1 According to these calculations, there is between a twofold and threefold reduction of NGAME at the lower growth rate range. Notably, these results are in agreement with Rebnegger *et al*. (2016), who reported threefold reduction in the maintenance requirements at low growth rates.

**Figure 5:**
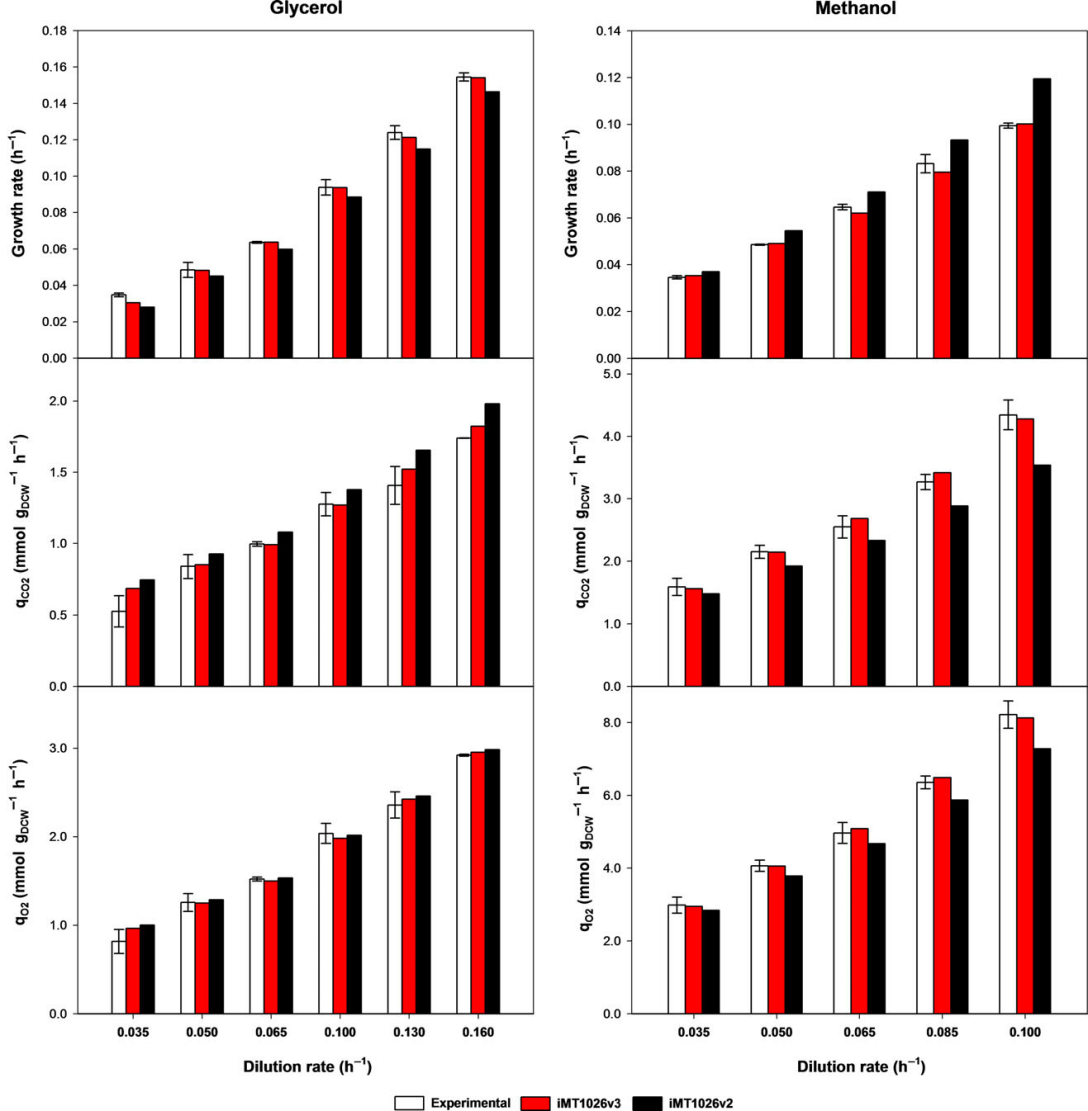
Performance of iMT1026 v3.0 and iMT1026 v2.0 models compared to experimental data for the glycerol and methanol cultivations at different growth rates. For the simulations, the specific substrate uptake rate was set as constraint, and biomass was maximized. In iMT1026 v3.0, the specific biomass equations, as well as new non‐growth associated maintenance energy values for glycerol and methanol, were enabled accordingly to the corresponding carbon source‐specific simulation.

Compared to iMT1026 v2.0, this new version improves the accuracy in the prediction of the main macroscopic variables for glycerol‐ or methanol‐grown biomass (Fig. 5) In addition, To demonstrate the importance of using accurate NGAME and GAME as well as precise condition‐specific biomass composition equations, a series of simulations were performed by changing each one of NGAME, GAME and biomass equations, and its overall accuracy was compared (Table S3 in Appendix S1). Results showed the best accuracy when all the parameters were adjusted to each specific carbon source. Thus, simulations using glycerol‐ or methanol‐specific biomass equations and estimated NGAME and GAME showed an overall deviation around 2%, while those simulations of glycerol and methanol cultivation data using the glucose‐specific biomass equation and energetic parameters resulted in average deviations of 7–10%. The isolated adjustment of one of the two energetic parameters to those calculated in iMT1026 v3.0 does not result in a significant increase in overall accuracy in all the cases. Despite improvements can be observed when predicting glycerol cultivation data with adjusted both GAME and NGAME parameters when methanol cultivation data are simulated, no such improvement is observed by changing only one of the two parameters. However, the setting of both GAME and NGAME values to those calculated specifically for glycerol and methanol has a positive effect on model prediction accuracy for both carbon sources, and model prediction deviations are reduced to 3–5%. Finally, an alternative approach was tested using the glucose‐specific biomass equation and recalibrating the GAME values, as described in ‘Energetic parameters calculation’ section in Experimental Procedures and according to the experimental values for glycerol and methanol cultivations. As reported in Table S3, simulations performed with these recalibrated GAME values are able to reduce the deviation of estimated values from experimental data (2–4%), but discrepancy still remained over the deviation of iMT1026 v3.0 that uses specific energetic parameters and macromolecular biomass equations. Hence, the calculation of new non‐growth associated maintenance energy coefficient according to Pirt's equation (Pirt, 1982) and the subsequent recalibration of growth associated maintenance energy might be an alternative for adapting genome‐scale metabolic models to expand the model to other carbon sources. Thus even without having new carbon source‐specific biomass macromolecular compositions, a GSMMs could be used for simulations with alternative carbon sources. However, such approach implies a penalty in overall prediction accuracy; thus, it could be used assuming higher deviations (more than twofold higher in the glycerol example, Table S3). Moreover, despite achieving acceptable macroscopic parameter estimations (2–5%) without adapting the biomass composition, an inaccurate description of biomass composition may result in false predictions of gene or enzyme essentiality (Duarte *et al*., 2004). In addition, metabolic flux distribution is sensitive to biomass composition (Dikicioglu *et al*., 2015); therefore, a wrong or inaccurate biomass equation may result in the estimation of erroneous flux distributions. Therefore, adapting the biomass equations to the condition‐specific composition would be the more accurate approach that would reflect the *in vivo* flux distribution with greater accuracy.

## Conclusions

In this study, we analysed the performance of *P. pastoris* growing in chemostat cultures using glycerol or methanol as single carbon source over a wide range of growth rates. The observed biomass composition changes in terms of protein and RNA content as a function of growth rate further supports the growth rate effect hypothesis on biomass composition; i.e. for both carbon sources, higher content of protein and RNA was observed at higher growth rates. Moreover, biomass composition also showed a strong dependence on carbon source, as protein content in biomass was higher in methanol‐grown cells. In addition, the carbon source has a significant impact on lipid and amino acid profiles.

Overall, the information gathered on biomass composition at different growth rates and carbon sources allowed to calculate average biomass compositions for glycerol‐ and methanol‐grown biomass. This allowed us to extend the iMT1026 model with new biomass equations for growth on glycerol or methanol as sole carbon sources. Energetic maintenance requirements were estimated for the first time in *P. pastoris* in both carbon sources. Furthermore, the model was validated for the range of growth rates tested, and it accurately described the experimental physiological data. Minor discrepancies between experimental data and simulations were found for glycerol at lower growth rates, where a nonlinear behaviour of growth parameters has been reported due to a metabolic shift on metabolism that enables *P. pastoris* to reduce its maintenance energy requirements. Such discrepancies can be easily taken into account by decreasing the value of maintenance energy requirements included in the model. Experimental data derived from chemostat cultivations provide information for calculating carbon source‐specific energetic parameters. These values allow for significantly improving the precision of estimated macroscopic behaviour. Therefore, the recalibration of energetic parameters, both NGAME and GAME, may be used for extending the model to alternative carbon sources. Furthermore, the characterization of biomass and definition of condition‐specific biomass equations enhance model performance and accuracy and allow for a more precise and realistic calculation of metabolic flux distribution.

In summary, the third version of iMT1026, v3.0, consensus model for *P. pastoris,* provides to the scientific community an improved metabolic engineering and analysis tool with expanded capabilities for predicting the metabolic phenotype in a broader range of conditions as well as an improved tool for future design of model‐based metabolic engineering of the *P. pastoris* cell factory.

## Experimental procedures

### Strain and cultivation conditions

*Pichia pastoris* wild‐type X‐33 (Invitrogen – Thermo Fisher Scientific, Carlsbad, CA, USA) was cultivated in carbon source‐limited chemostat cultures at a range of dilution rates. Continuous cultures were performed at a working volume of 1 l in a 2 l benchtop bioreactor Biostat B (Sartorius AG, Göttingen, Germany) for glycerol cultures and in a Biostat B+ (Sartorius AG) for methanol cultivations. Two independent chemostat series were performed for each carbon source of increasing dilution rates (D) of 0.035, 0.050, 0.065, 0.100, 0.130 and 0.160 h^−1^ for glycerol and 0.035, 0.050, 0.065, 0.080, 0.100 and 0.130 h^−1^ for methanol. For preculture, 150 ml of YPG media (2% (w/v) peptone, 1% (w/v) yeast extract and 2% (w/v) glycerol) in 1 l of shake flasks was inoculated with a cryostock at an initial OD_600_ of 0.15–0.30 and incubated at 150 rpm and 25°C (Infors HT Multitron, Bottmingen, Switzerland) for approximately 24 h. Cells were centrifuged and resuspended in sterile demineralized water and used to inoculate the bioreactor for the batch phase. Once the batch phase was concluded, chemostat phase was initiated at the specific growth rate by appropriately setting the corresponding inlet flow and enabling outlet flow to keep the reactor volume constant to 1 l. Both for batch and chemostat culture, stirring was set to 700 rpm, aeration rate to 1 vvm, temperature was maintained at 25°C and pH 5.0 automatically controlled with 15% ammonia. The off‐gases were cooled dawn in a condenser at 4°C and further desiccated in two silica gel columns. For the glycerol cultures, off‐gas CO_2_ and O_2_ fractions were analysed through BCP‐CO_2_ and BCP‐O_2_ Sensors (BlueSens gas sensor GmbH, Herten, Germany). On the other hand, methanol off‐gas composition was analysed by means of a mass spectrometer Omnistar™ 300 02 (Balzers Instruments, Balzers, Liechtenstein). Each dilution rate was kept for at least five residence times, and three culture samples were taken along the last volume change.

Batch medium composition was previously described in Baumann *et al*. (2008). Chemostat medium composition was also taken from Baumann *et al*. (2008), except that glucose was replaced by glycerol or methanol as carbon source. Thus, briefly chemostat medium contained per litre: 50 g carbon source (glycerol or methanol), 0.84 g citric acid, 4.35 g (NH4)_2_HPO_4_, 0.01 g CaCl_2_ · 2H_2_O, 1.7 g KCl, 0.65 g MgSO_4_ · 7H_2_O, 1 ml Biotin (0.2 g l^−1^) and 1.6 ml PTM1 trace salts stock solution (prepared as described in Baumann *et al*., 2008). pH was adjusted to 5.0 with 25% HCl.

### Analytical methods

#### Extracellular metabolite quantification

Glycerol, methanol, arabitol, succinate, acetate and ethanol were analysed by HPLC. Triplicate samples (2 ml each) were centrifuged at 12 000 rpm for 2 min (Minispin, Eppendorf, Hamburg, Germany). The supernatant was collected and filtered through 0.45 μm nitrocellulose membrane filters (Merck Millipore, Carrigtwohill, Ireland). Duplicate samples were analysed by HPLC (HP 1050 liquid chromatograph, Dionex Corporation, Sunnyvale, CA, USA) using an ICSep ICE COREGEL 87H3 column (Transgenomic Inc., Omaha, NE, USA). The mobile phase was 8 mM sulphuric acid. Injection volume was 20 μl. Data were quantified by Chromeleon 6.80 Software (Dionex Corporation, Sunnyvale, CA, USA). Average relative standard deviation (RSD) of the analysis was about 1%.

#### Biomass quantification

Biomass in culture broth was monitored during cultivation by measuring the optical density at 600 nm. Dry cell weight (DCW) was quantified accordingly to the method described in Jordà *et al*. (2012). Biomass concentration was determined in triplicate. Biomass concentration average RSD was about 2%.

#### Biomass composition analysis

Both for the glycerol and methanol cultivations, biomass composition was analysed at the following growth rates: 0.035, 0.065, 0.100 h^−1^. Additionally, for the glycerol cultivations, biomass analyses were also carried out at 0.160 h^−1^.

##### Elemental analysis

C, H, N, S were analysed by combustion at 1200°C and subsequent gas chromatography in a Flash 2000 Elemental Analyzer (Thermo Fisher Scientific, Waltham, MA, USA). Oxygen was determined through an oxygen‐specific pyrolysis at 1060°C. Ash content was determined by subtraction of the C, H, N, O, S fractions as remaining component.

##### Amino acid analysis

Fifteen milligrams of lyophilized biomass was hydrolysed with 6M HCl for 24 h at 105°C. Then, deionized water (MiliQ) was added up to complete 50 ml. Filtered aliquots were vacuum‐dried and finally resuspended in water. Samples were then derivatized wit 6‐aminoquinolyl‐N‐hydroxysuccinimidyl carbamate according to AccQ‐Tag method (Waters, Milford, MA USA). Derivatized amino acids were analysed with a Waters 2487 (Waters) UV detector at 254 nm in a gradient system HPLC Waters 600 (Waters).

Biomass samples for the determination of total protein, carbohydrates, DNA and RNA content were prepared and analysed as described in Carnicer *et al*. (2009).

### Statistical analysis

Standard reconciliation procedures (Lange and Heijnen, 2001; Verheijen, 2010) were applied to elemental composition and major macromolecular components (proteins, carbohydrates, DNA and RNA). The resulting biomass elemental composition was subsequently used to perform chemostat cultivation data reconciliation and consistency analyses (Noorman *et al*., 2000). Both for biomass macromolecular and elemental composition as well as for chemostat substrate and product data, a statistical consistency test, based on h‐index as described by Noorman *et al*. (2000) was passed with a confidence level of 95%. Consequently, there was no evidence for gross measurement errors.

Global macromolecular, amino acid and lipid composition data were analysed with statistical tests available in Microsoft Excel. Two‐tailed Student's *t*‐test was used to determine statistically significant differences in biomass composition between carbon source and growth rates.

### Modelling

*Pichia pastoris* iMT1026 v2.0 (Tomàs‐Gamisans *et al*., 2016) updated at BioModels database (Chelliah *et al*., 2015) ID: MODEL1508040001 (Appendix S2) was used as starting model for further updating. The model was edited incorporating new average carbon source‐specific biomass equations using standard scripts from cobra toolbox v2.0.6 (Schellenberger *et al*., 2011). Appendix S4 includes the cobra commands necessary to add these new equations into the existing model. The biomass stoichiometric coefficients are directly derived from the carbon source‐specific average biomass composition determined experimentally and summarized in Table 2. All simulations were carried out with the cobra toolbox v2.0.6 under Matlab 2014 (Mathworks, Natick, MA, USA) with sbml toolbox v4.1.0 (Keating *et al*., 2006) and libsbml library v5.12.0 (Bornstein *et al*., 2008). Flux balance analysis (FBA) with linear optimization was used to predict metabolic phenotypes by setting the appropriate flux constraints. To test model accuracy and validate it for each carbon source, biomass production was constrained to each of the experimentally tested growth rates, and the absolute value of substrate uptake rate was minimized performing a FBA. The resulting macroscopic fluxes (O_2_ and substrate consumption and CO_2_ production) were calculated and compared with the corresponding experimental values. iMT1026 v3.0 model was saved in SBML format, validated for syntax and internal consistency and submitted to BioModels database with the ID: MODEL1612130000. This model is also available in Appendix S3

#### Energetic parameters calculation

ATP requirement for cellular maintenance was determined by the following energetic parameters estimation procedure. These requirements were divided into growth associated maintenance energy (GAME) and non‐growth associated maintenance energy (NGAME). For NGAME calculation, the substrate uptake rate was represented against the growth rate (μ) according to Pirt's equation (Pirt, 1982). In the *y‐*intercept of this linear regression, ATP turnover was maximized (μ = 0). These ATP values (for glycerol and methanol) are set as lower bounds in ‘ATPM’ reaction, representing NGAME.

Using the obtained values for NGAME, GAME was determined by adjusting ATP stoichiometric coefficient in the corresponding biomass equation to fit biomass–substrate yields according to the experimental data. These simulations were carried out by maximizing the biomass production in a FBA, at the different growth rates, constraining the substrate uptake rate according to the experimental data and iteratively fitting the ATP stoichiometric coefficient to the less global residual error of predicted biomass to the experimental values.

## Conflict of interest

None declared.

## Supporting information


**Appendix S1**
**Table S1.** Amino acid composition of cell protein extracts for all the growth conditions tested. Values represent % mol/mol ± SD.
**Table S2.** Biomass lipid profile in all the tested conditions. Values represent % w/w of the lipid fraction ± SD.
**Table S3.** Evaluation of macroscopic parameter prediction accuracy using different energetic parameters and biomass composition configurations.
**Fig. S1.** Prediction of macroscopic growth parameters in glycerol‐grown cells at 0.035 h^−1^ using different values for non‐growth associated maintenance (ATPM). Substrate uptake rate was constrained according to the experimental data and different values for the ‘ATPM’ reaction were tested. Default ATPM corresponding to glycerol‐grown biomass is 2.9 mmol ATP·g_DCW_^−1^·h^−1^. q_CO2_: experimental (solid line) and predicted (●); growth rate: experimental (dotted line) and predicted (□); q_O2_: experimental (dashed line) and predicted (▼).

**Appendix S2** iMT1026v2.xml. Second version of iMT1026 (v2.0) model in SBML format.

**Appendix S3.** iMT1026v3.xml. New updated version of iMT1026 (v3.0) in SBML format.

**Appendix S4.** iMTv3Edition.txt. COBRA commands applied to iMT1026 v2.0 for the generation of iMT1026 v3.0.
