# Supplementary material for: Fine‐tuning the P. pastoris iMT1026 genome‐scale metabolic model for improved prediction of growth on methanol or glycerol as sole carbon sources
Source: Microb Biotechnol. 2017 Nov 21;11(1):224–37. doi: 10.1111/1751-7915.12871 (PMC5743807; doi:10.1111/1751-7915.12871)
Supplement: Supplementary file 1 — Appendix S1 Table S1. Amino acid composition of cell protein extracts for all the growth conditions tested. Values represent % mol/mol ± SD. Table S2. Biomass lipid profile in all the tested conditions. Values represent % w/w of the lipid fraction ± SD. Table S3. Evaluation of macroscopic parameter prediction accuracy using different energetic parameters and biomass composition configurations. Fig. S1. Prediction of macroscopic growth parameters in glycerol‐grown cells at 0.035 h−1 using different values for non‐growth associated maintenance (ATPM). Substrate uptake rate was constrained according to the experimental data and different values for the ‘ATPM’ reaction were tested. Default ATPM corresponding to glycerol‐grown biomass is 2.9 mmol ATP·gDCW −1·h−1. qCO2: experimental (solid line) and predicted (●); growth rate: experimental (dotted line) and predicted (□); qO2: experimental (dashed line) and predicted (▼). [file MBT2-11-224-s001.pdf]

Appendix S1

Fine-tuning the *Pichia pastoris* iMT1026 genome-scale metabolic model for improved prediction of growth on methanol or glycerol as sole carbon sources.

Màrius Tomàs-Gamisans, Pau Ferrer and Joan Albiol

In this supporting file, additional details on the biomass composition are provided. Supporting **Tables S1–2** show specific biomass composition for all the conditions tested. The reconciled macromolecular and elemental composition (**Table 2**) is included in the main article, amino acid composition (**Table S1**) and lipid profile of each condition (**Table S2**).

These tables also include the average compositions for glycerol and methanol used for building the new biomass equations in iMT1026 v3 and detailed in **Appendix S4**.

For a better comparison of the impact of the GAME and NGAME values, the new model with new biomass equations was compared to simulations performed with the glucose biomass equation and new and recalibrated values for GAME and NGAME (**Table S3**)

The reduction of maintenance energy requirements allowed predicting macroscopic growth parameters using iMT1026 v3.0 (**Fig S1**).

Table S1. Amino acid composition of cell protein extracts for all the growth conditions tested. Values represent % mol/mol ± sd.

D (h^{-1})	Glycerol				Methanol			Average glycerol [c]	Average methanol [c]	*p* -value [d]
	**0.035**	**0.065**	**0.100**	**0.160**	**0.035**	**0.065**	**0.100**			
**Ala**	9.0 ± 0.8	8.7 ± 0.7	9.2 ± 0.3	9.9 ± 0.09	8.2 ± 0.1	8.1 ± 0.7	8.9 ± 0.8	9.0 ± 0.5	8.5 ± 0.5	0.045
**Arg**	5.12 ± 1.4	4.8 ± 0.5	6.1 ± 1.8	5.3 ± 0.90	5.5 ± 3e-2	5.6 ± 0.2	5.6 ± 0.6	5.5 ± 0.6	5.6 ± 0.1	0.548
**Asx** [a]	10.3 ± 2.0	10.2 ± 0.2	9.4 ± 1.3	9.6 ± 4e-3	12.0 ± 0.1	11.9 ± 0.2	11.9 ± 1.5	10.0 ± 0.4	11.9 ± 0.1	0.001
**Cys**	0.4 ± 0.1	0.4 ± 0.2	0.2 ± 1e-2	0.3 ± 0.1	0.4 ± 0.3	0.3 ± 0.1	0.4 ± 0.2	0.4 ± 0.1	0.4 ± 0.1	0.510
**Glx** [a]	16.2 ± 2.6	14.4 ± 0.4	16.5 ± 2.6	15.2 ± 0.3	13.3 ± 0.8	13.5 ± 0.5	13.9 ± 0.5	16.2 ± 1.0	13.5 ± 0.3	0.012
**Gly**	5.9 ± 1.1	7.8 ± 0.1	7.3 ± 0.6	7.7 ± 3e-2	7.4 ± 0.7	7.5 ± 0.7	6.8 ± 2.0	6.5 ± 0.9	7.1 ± 0.4	0.926
**His**	2.1 ± 0.1	2.1 ± 0.1	2.1 ± 0.1	2.1 ± 0.1	2.5 ± 0.1	2.5 ± 0.1	2.2 ± 2e-2	2.1 ± 3e-2	2.5 ± 0.2	0.005
**Ile**	4.5 ± 0.6	4.6 ± 0.1	4.4 ± 0.5	4.9 ± 0.2	4.7 ± 0.2	4.9 ± 0.4	4.7 ± 0.1	4.6 ± 0.2	4.8 ± 0.1	0.289
**Leu**	7.1 ± 1.1	7.7 ± 0.6	7.1 ± 0.1	7.4 ± 0.5	7.7 ± 0.2	7.7 ± 0.1	7.7 ± 0.1	7.3 ± 0.3	7.7 ± 3e-2	0.116
**Lys**	7.0 ± 1.0	6.9 ± 1e-2	6.6 ± 0.4	6.7 ± 0.2	6.7 ± 0.2	6.7 ± 0.1	7.1 ± 0.4	6.9 ± 0.2	7.0 ± 0.3	0.894
**Met**	1.0 ± 0.2	1.0 ± 0.1	0.6 ± 0.3	0.9 ± 0.2	1.0 ± 0.3	1.0 ± 0.1	1.0 ± 0.1	0.8 ± 0.2	1.00 ± 3e-2	0.253
**Phe**	3.2 ± 0.5	3.5 ± 0.1	3.3 ± 0.3	3.3 ± 0.1	3.4 ± 0.1	3.4 ± 4e-2	3.5 ± 0.1	3.3 ± 0.1	3.5 ± 2e-2	0.155
**Pro**	4.7 ± 0. 1	4.6 ± 0.2	4.5 ± 0.1	4.3 ± 0.1	4.7 ± 3e-2	4.8 ± 3e-2	4.3 ± 0.1	4.5 ± 0.2	4.5 ± 0.3	0.555
**Ser**	6.8 ± 0.1	7.0 ± 0.3	6.6 ± 0.3	6.6 ± 0.1	6.4 ± 0.1	6.4 ± 0.2	6.2 ± 0.2	6.8 ± 0.2	6.3 ± 0.1	0.002
**Thr**	7.3 ± 0.2	6.8 ± 0.2	6.9 ± 0.2	6.5 ± 0.2	6.4 ± 0.3	6.4 ± 0.1	6.1 ± 0.1	6.9 ± 0.4	6.3 ± 0.2	0.003
**Trp** [b]	1.2 ± n.d.	1.0 ± n.d.	1.1 ± n.d.	0.99 ± n.d.	1.0 ± n.d.	0.8 ± n.d.	0.9 ± n.d.	1.1 ± 0.1	0.9 ± 0.1	0.040
**Tyr**	2.3 ± 0.3	2.4 ± 0.1	2.1 ± 0.1	2.3 ± 0.1	2.8 ± 0.1	2.6 ± 0.1	2.6 ± 0.1	2.3 ± 0.1	2.6 ± 0.1	2.4e-4
**Val**	6.0 ± 0.7	6.4 ± 0.1	5.9 ± 0.4	6.1 ± 3e-2	5.9 ± 4e-2	5.9 ± 0.1	6.1 ± 0.1	6.0 ± 0.2	6.0 ± 0.1	0.424

[a] Asx and Glx represent the pair of Asp/Asn and Glu/Gln respectively.
[b] Trp was not measured, thus, recalculated according to values in Carnicer *et al.* (2009).
[c] Average compositions for each carbon source are weighted averages using 1/sd of the different analysed growth rates.
[d] *p*-value resulting of the comparison of glycerol and methanol datasets for each amino acid by applying a 2-tailed Student's t-Test. Statistically significant differences on composition were considered when *p*-value < 0.05.

Table S2. Biomass lipid profile in all the tested conditions. Values represent % w/w of the lipid fraction ± sd.

	Glycerol				Methanol			Average glycerol [b]	Average methanol [b]
**D (h^{-1})**	**0.035**	**0.065**	**0.100**	**0.16**	**0.035**	**0.065**	**0.100**		
**TAG**	7.5 ± 2.5	5.9 ± 1.7	9.4 ± 4.7	6.8 ± 4.99	0.1 ± 0.1	n.d.	n.d.	7.1 (6.9) ± 1.5	n.d.
**FFA**	36.1 ± 8.8	27.7 ± 1.3	28.8 ± 1.1	21.3 ± 4.54	53.7 ± 1.3	42.4 ± 2.8	36.2 ± 14.9	28.4 (27.4) ± 6.0	46.4 (44.7) ± 8.9
**STE**	20.4 ± 1.6	16.6 ± 5.7	18.5 ± 0.6	23.0 ± 3.2	16.3 ± 6.2	17.7 ± 2.8	19.5 ± 9.5	19.6 (18.9) ± 2.7	16.6 (16.0) ± 1.6
**PE**	11.3 ± 7.2	14.0 ± 1.0	12.0 ± 3.4	12.0 ± 0.3	6.9 ± 1.7	10.7 ± 3e-3	11.8 ± 1.5	12.6 (12.2) ± 1.2	10.0 (9.6) ± 2.6
**CAR**	2.7 ± 1.6	3.6 ± 0.5	2.8 ± 1.4	2.2 ± 0.5	1.7 ± 1.0	2.6 ± 0.5	3.2 ± 0.2	2.9 (2.8) ± 0.6	2.7 (2.6) ± 0.7
**PA**	0.5 ± 0.7	1.6 ± 0.6	0.4 ± 0.5	1.1 ± 1e-3	0.4 ± 0.6	0.3 ± 0.4	0.3 ± 0.4	1.1 (1.1) ± 0.6	0.3 (0.3) ± 0.1
**PC**	15.3 ± 4.2	19.1 ± 1.6	17.3 ± 2.0	19.9 ± 4.3	10.5 ± 4.0	17.3 ± 0.9	17.1 ± 4.3	18.4 (17.7) ± 2.1	15.2 (14.7) ± 3.9
**PI/PS**	6.4 ± 0.8	11.4 ± 3.9	10.9 ± 1.8	13.7 ± 1.3	10.4 ± 0.1	9.0 ± 4e-2	12.0 ± 0.6	9.9 (9.5) ± 3.1	8.9 (8.6) ± 1.5
**SPH** [a]								(3.6)	(3.6)

TAG: Triacylglyceorls; FFA: free fatty acids; STE: sterols; PE: phosphatidylethanolamine CAR: cardiolipin; PA: phosphatidic acid; PC: phosphatidylcholine; PI/PS: phosphatidylinositol / phosphatidylserine; SPH: sphingolipids.

[a] SPH content was not measured in lipid analysis. Thus, as well as in Tomàs-Gamisans *et al.* (2016), values are taken from Grillitsch *et al.* (2014).

[b] Average compositions are weighted averages using 1/sd. In brackets, values used in iMT1026 v3 and represent the rescaled lipid content with the addition of the SPH fraction.

Impact of biomass equation and energetic parameters on model accuracy

Models with different energetic parameters and biomass compositions were compared by performing a Flux Balance Analysis, maximising the biomass production equation and constraining the substrate uptake rate according to the experimental values. Macroscopic parameters (q_X, q_{CO2}, q_{O2}) were compared against the experimental values and evaluated using the statistical parameter described in eq. 1.

$$\frac{1}{n} \cdot \sum_{i=1}^{n} \frac{\sqrt{\left(v_{s_i} - v_{e_i}\right)^2}}{v_{e_i}} \% \qquad (eq\ 1)$$

where v_{s_i} is the resulting flux from the simulation for the variable i and v_{e_i} is the experimental flux for this variable. The total number of predicted and compared fluxes with the experimental values is n and equals to 15.

Table S3 ***Evaluation of macroscopic parameter prediction accuracy using different energetic parameters and biomass composition configurations*.** Prediction accuracy was calculated using eq. 1 with the corresponding simulation and experimental values. iMT1026v3 corresponds to the results using new model with the carbon source specific biomass compositions. Glucose BM equation indicates the simulation results using iMT1026 v3.0 with the glucose biomass equation (original) and those GAME and NGAME associated to the glucose (72.1 and 2.91 respectively). The other simulations were performed all with the glucose specific biomass composition and changing the energetic parameters described as follows: + NGAME, indicates the adjustment of NGAME to that corresponding to the glycerol or methanol case (2.51 and 0.44 respectively); + GAME corresponds to the simulations using the glucose-specific biomass equation with the GAME coefficients corresponding to those in iMT1026 v3.0 for each carbon source. +NGAME +GAME shows the resulting accuracy of changing both GAME and NGAME to the carbon-source specific values while using the glucose-specific biomass equation. Finally, recalculated GAME, corresponds to a new recalibration of GAME coefficients using the glycerol and methanol NGAME and the glucose-specific biomass equation.

	Glycerol	Methanol
**iMT1026 v3.0**	1.80%	2.15%
**Glucose BM equation**	6.87%	10.22%
**+NGAME**	5.19%	20.56%
**+GAME**	6.19%	10.28%
**+NGAME +GAME**	4.68%	2.83%
**Recalculated GAME**	4.24%	2.33%

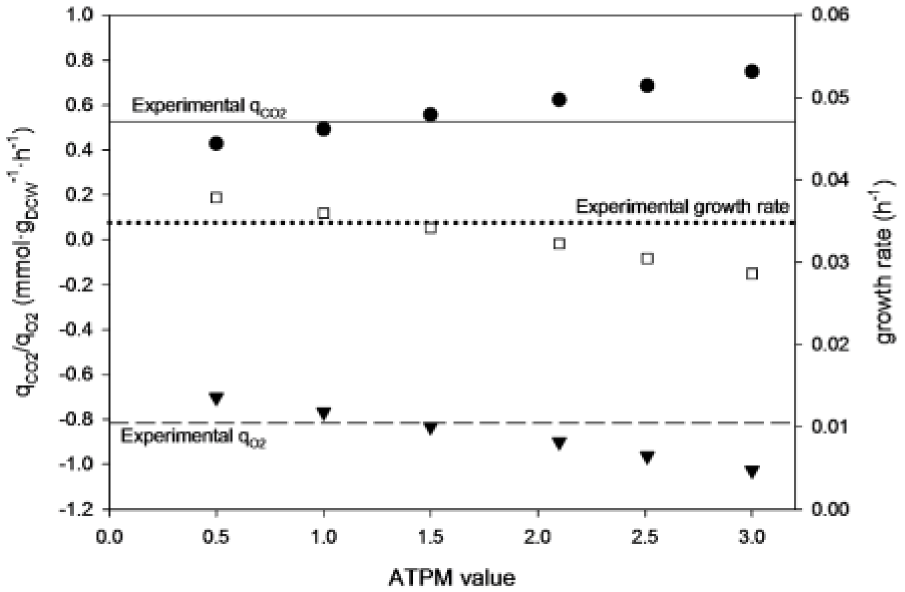

Fig. S1. *Prediction of macroscopic growth parameters in glycerol-grown cells at 0.035 h^{-1} using different values for non-growth associated maintenance (NGAME). A Flux Balance Analysis ws performed maximizing growth rate and constraining the substrate uptake rate according to the experimental values. Different values for the 'ATPM' reaction (representing NGAME) were tested from 0 to 3. The default ATPM corresponding to glycerol-grown biomass is 2.51 mmol ATP·g_{DCW}^{-1}·h^{-1}. In the figure, the main experimental and estimated macroscopic parameters are represented: experimental q_{CO2} (solid line) and estimated q_{CO2} (●); experimental growth rate (dotted line) and estimated growth rate (□); experimental q_{O2} (dashed line) and estimated q_{O2} (▼).*

References

Carnicer, M., Baumann, K., Töplitz, I., Sánchez-Ferrando, F., Mattanovich, D., Ferrer, P., and Albiol, J. (2009) Macromolecular and elemental composition analysis and extracellular metabolite balances of *Pichia pastoris* growing at different oxygen levels. *Microb. Cell Fact.* **8**: 65.

Grillitsch, K., Tarazona, P., Klug, L., Wriessnegger, T., Zellnig, G., Leitner, E., et al. (2014) Isolation and characterization of the plasma membrane from the yeast *Pichia pastoris*. *Biochim. Biophys. Acta - Biomembr.* **1838**: 1889–1897.

Tomàs-Gamisans, M., Ferrer, P., and Albiol, J. (2016) Integration and Validation of the Genome-Scale Metabolic Models of *Pichia pastoris*: A Comprehensive Update of Protein Glycosylation Pathways, Lipid and Energy Metabolism. *PLoS One* **11**: e0148031.
